# Renal and neurological side effects of colistin in critically ill patients

**DOI:** 10.1186/2110-5820-1-14

**Published:** 2011-05-25

**Authors:** Herbert Spapen, Rita Jacobs, Viola Van Gorp, Joris Troubleyn, Patrick M Honoré

**Affiliations:** 1 Intensive Care Department, University Hospital, Vrije Universiteit Brussel, Laarbeeklaan 101, B-1090 Brussels, Belgium

## Abstract

Colistin is a complex polypeptide antibiotic composed mainly of colistin A and B. It was abandoned from clinical use in the 1970s because of significant renal and, to a lesser extent, neurological toxicity. Actually, colistin is increasingly put forward as salvage or even first-line treatment for severe multidrug-resistant, Gram-negative bacterial infections, particularly in the intensive care setting. We reviewed the most recent literature on colistin treatment, focusing on efficacy and toxicity issues. The method used for literature search was based on a PubMed retrieval using very precise criteria.

Despite large variations in dose and duration, colistin treatment produces relatively high clinical cure rates. Colistin is potentially nephrotoxic but currently used criteria tend to overestimate the incidence of kidney injury. Nephrotoxicity independently predicts fewer cures of infection and increased mortality. Total cumulative colistin dose is associated with kidney damage, suggesting that shortening of treatment duration could decrease the incidence of nephrotoxicity. Factors that may enhance colistin nephrotoxicity (i.e., shock, hypoalbuminemia, concomitant use of potentially nephrotoxic drugs) must be combated or controlled. Neurotoxicity does not seem to be a major issue during colistin treatment. A better knowledge of colistin pharmacokinetics in critically ill patients is imperative for obtaining colistin dosing regimens that ensure maximal antibacterial activity at minimal toxicity.

## Introduction

Colistin belongs to the polymyxin class of cationic polypeptide antibiotics. It is administered as the prodrug colistimethate sodium (CMS), a fraction of which is hydrolyzed *in vivo *to colistin. During the 1970s, the popularity of colistin rapidly faded because of reports of significant renal and neurological toxicity and it was progressively supplanted by less toxic antibiotics with a comparable or broader antibacterial spectrum. However, the mounting prevalence worldwide of infections due to multidrug-resistant (MDR) Gram-negative bacilli has renewed interest into colistin but also revived the discussion about its toxicity [1].

We searched the PubMed database for English language studies (a) published during the last 15 years (from January 1995 to December 2010); (b) including at least 10 critically ill adult patients without cystic fibrosis treated with intravenous CMS as primary or salvage therapy for MDR Gram-negative organisms; and (c) reporting data on efficacy, nephrotoxicity, and neurotoxicity. A total of 26 relevant studies [2-27] were identified and are summarized in Table 1.

**Table 1:** Dosage, duration, outcome, and toxicity of intravenous colistimethate sodium in critically ill patients

Author	Patients (N)	APACHE II (mean ± SD)	CMS dose/duration [mean ± SD or median (range)]	Clinical cure N (%)	Nephrotoxicity N (%)	Neurotoxicity
Levin	59 (60 infections)	13.1 ± 7	152.8 mg ± 62.8 mg 12.6 ± 6.8 days	35 (58.3)	22 (37)	none
Markou	24 (26 infections)	20.6 (mean)	3 MIUq8h 13.5 days (4-24 days)	17 (65.4)	3 (14.5)	none
Garnacho- Montero	21	19.6 ± 7.2	2.5 mg-5 mg/kg/day 14.7 ± 4.1days	12 (57.1)	5 (24)	none
Michalopoulos	43	25.8 ± 7.7	3 MIUq8h 18.6 ± 5.8 days	32 (74)	8 (18.6)	none
Falagas	17 (19 infections)	14 (median) 43.4 ± 14.6 days	4.4 MIU ± 2.1 MIU	14 (74)	1 (5.2)	1
Kasiakou	50 (54 infections)	16.1 ± 6.1	4.5 MIU ± 2.3 MIU 21.3 ± 16 days	36 (66.7)	4 (8)	1^**a**^
Reina	55	21 ± 7	5 mg/kg (max 300 mg/day) 13 ± 5 days	NA	0 (0)	none
Petrosillo^**b**^	14	NA	2 MIUq8h 12 days (mean)	9 (64)	1 (7.1)	none
Kallel	75 (78 infections)	NA (SAPS II 37 ± 14)		5.5 MIU ± 1.1 MIU 9.3 ± 3.8 days	60 (76.9) 7/52 (13.5)	1
Koomanachai	78	21.9 (mean)	179.6 mg/day (mean) 11.9 days (mean)	63 (80.8)	24 (30.8)	none
Betrosian	15	14 ± 2	5.83 MIU ± 2.3 MIU duration NA	9 (60)	5 (33)	none
Bassetti^**b**^	29	17 ± 3.7	2 MIUq8h 17.7 ± 10.4 days	22 (76)	3 (10)	none
Kallel	60	NA (SAPS II 35 ± 12)	2 MIUq8h 9.5 ± 3.8 days	45 (75)	0 (0)	NA
Falagas	21	19 ± 4	5.5 MIU ± 1.9 MIU 17.7 ± 11.7 days	11 (52.4)	3 (14.3)	none
Falagas	14 (CMS mono)	14.3 ± 7.4	4.6 MIU ± 2.3 MIU 14.2 ± 7.3 days	12 (85.7)	0 (0)	NA
	57 (CMS+MERO)	15.4 ± 6.6	5.5 MIU ± 2.2 MIU 17.8 ± 11.4 days	39 (68.4)	4 (7)	NA
Pintado	60	11.2 ± 7.7	4.42 MIU ± 1.39 MIU 20 ± 9.2 days	43 (71.7)	6/55 (10.9)	none
Sabuda	12	NA	3.7 mg/kg 14.7 ± 13.8 days	8 (66.7)	5 (41.6)	4 cases
Huang	15	14.7 ± 4.5	1.28 MIU ± 0.25 MIU 22.3 ± 6.2 days	11 (73.3)	0 (0)	none
Hartzell	66	8.3 ± 6.5	4.3 ± 1.2 mg/kg/day 15.8 ± 9.2 days	NA	30 (45)	2 cases
Kim	42 (47 infections)	NA	2.25 g (0.6-8.7g)^**c**^ 16.6 ± 14.8 days^**e**^	10/15 (66)^**d**^	15 (31.9)	none
Kwon	71	NA	4.6 mg/kg (median) 13 days (7-22 days)	NA	38 (53.5)	none
Cheng	115^**f**^	6 (median)	dose NA 12 ± 7days^**g**^	59 (51)	12/84 (14)	4 cases
Song	10	NA	150 mg q12h 8.1 ± 1.8 days	7 (70)	0 (0)	none
Falagas^**h**^	258	17 (range 2-39)	up to 3 MIUq8h mean 17.9 days (10-22)	204 (79.1)	26 (10)	NA
Kofteridis	43	17.7 ±7.6	3 MIUq8h median 10 days (4-36 days)	14 (32.5)	8 (19)	none
DeRyke	30	13 (range 7-18)^**i**^	5.1 ± 2 mg/kg/day^**j**^ median 8 days (3-24)	NA	10 (33%)	none

NA = not available; APACHE II = Acute Physiology and Chronic Health Evaluation II score; SAPS 2 = Simplified Acute Physiology Score 2; MIU = million International Units; CMS = colistimethate sodium; MERO = meropenem.

^**a**^Same patient as in reference 6; ^**b**^+ rifampicine; ^**c**^mean cumulative CMS dose when nephrotoxicity occurred; ^**d**^in patients presenting nephrotoxicity; ^**e**^in patients presenting nephrotoxicity (vs. 9.5 ± 5.6 days in patients without nephrotoxicity, *p *= 0.07); ^**f**^31 patients on renal replacement therapy at start of CMS treatment; ^**g**^in patients with good clinical response (vs. 11 ± 11 days in patients with poor response); ^**h**^includes data on 108 patients from references 5, 7, and 16; ^**i**^in patients who developed nephrotoxicity; ^**j **^based on ideal body weight.

## Patient characteristics

The majority of patients were hospitalized in general or specialized intensive care units (ICU). Patients were mainly treated for pulmonary, catheter-related or primary bloodstream, urinary tract, (surgical) wound, and abdominal and central nervous system infections. The most frequently isolated pathogens were MDR *Acinetobacter baumannii *and *Pseudomonas aeruginosa*. Some overlap between studies is present. Kallel et al. evaluated CMS treatment in a wide array of infections [10] but also more specifically discussed a subgroup of patients with ventilator-associated pneumonia [14]. The large cohort study by Falagas et al. [25] included data on 108 patients, which were reported in previous studies from the same group [5,7,16]. Severity of illness, as determined by the APACHE II score, was very different between studies, which underline the individual impact of score-determining factors, such as age, pre-existing renal dysfunction, shock, and respiratory failure.

## Dose, duration, and efficacy of colistimethate sodium treatment

CMS was mostly administered for 10 to 14 days. Dose regimens varied considerably and were kept constant daily over time or adapted to the patients' weight. Doses were adjusted for renal function depending on serum creatinine levels or creatinine clearance. CMS was used as monotherapy, in association with synergistic antibiotics (e.g. rifampicin) or in combination with other broad-spectrum antimicrobials. Clinical efficacy and toxicity of CMS were evaluated regardless of whether CMS was prescribed as monotherapy or in combination with other agents. Global clinical cure for all infections taken together in all evaluable patients approached 70%.

The largest single-center cohort study to date retrospectively investigated 258 patients with microbiologically documented infection [25]. Patients were treated for at least 72 h with intravenous CMS, either alone or in combination with other antibiotics, and were evaluated during a 7-year period (2000-2007). Because few patients developed significant nephrotoxicity, the investigators progressively increased daily CMS doses over time, reaching a standardized protocol of 9 million IU per day (in 3 divided doses) during the last 2 years of the study. CMS dose was always adapted to renal function. Infection was cured in 79.1% of patients. Independent factors for a favorable infection outcome were antimicrobial regimens that consisted of CMS in monotherapy or in combination with meropenem (compared with CMS combined with other agents with potential activity against the isolated pathogen) and pneumonia (compared with bacteremia and abdominal infection), whereas age and proportional increase in creatinine independently predicted unfavorable infection outcome. Lower mean colistin daily dose (3 million IU compared with 6 and 9 million IU), APACHE II score, hematological disease, and nephrotoxicity were independent factors predicting increased mortality.

## Nephrotoxicity

Renal toxicity is the most common adverse effect of colistin treatment because the drug is excreted primarily by the kidneys and elevated blood levels may further impair renal function. Little information is available on the mechanism of toxicity but *in vitro *electrophysiological studies demonstrate that, at long exposure times, colistin is directly toxic to mammalian urothelium by increasing transepithelial conduction [28].

A disparity between old and recent studies exists in the reported rates of nephrotoxicity associated with intravenous administration of colistin [29]. The recent studies generally indicate a relatively lower incidence of renal toxicity. This can be explained by the use of more purified colistin, the use of colistimethate instead of colistin sulphate, more adequate dose adjustment according to renal function and significant improvement of ICU monitoring (in particular of the patient's hydration status) and treatment (more rapid and adequate resuscitation of severe sepsis and shock and avoidance of concomitant administration of potentially nephrotoxic agents).

Nephrotoxicity rates vary widely, ranging between 0% and 53.5% [2-27], but comparison between studies is hazardous and complicated by a lack of control for risk factors and a case mix of patients with and without renal dysfunction at baseline. Moreover, the wide range of reported nephrotoxicity rates probably reflects more the varying definitions of "renal failure" than the actual effect of colistin. In studies that discriminate between patients with normal and impaired renal function before start of colistin treatment, the incidence of nephrotoxicity was 2.5- to 7-fold higher in patients with baseline renal dysfunction [2,5,7]. Koomanachai et al. reported a 30.8% incidence of nephrotoxicity [11]. However, 70% of their patients had underlying or predisposing factors (chronic kidney disease, nephrotoxic drug use, hypovolemia), which might have contributed to a decline in renal function. Also, 58% of the patients were not treated in an ICU environment despite a high mean APACHE II score for the whole group. Nephrotoxic effects were mild and reversible, and no patient required renal replacement therapy. A Canadian study reported at least a doubling of serum creatinine in 5 of 12 patients (41.6%) treated with intravenous CMS for at least 3 days [18]. Once again, these patients had severe underlying diseases and comorbidities and all but one received at least one other potentially nephrotoxic drug. The only patient with renal failure admitted to the ICU even had received the equivalent of 13 million IU of colistin per day. It is noteworthy that the effectiveness and nephrotoxic potential of intravenous CMS was not different from imipenem in two studies comparing both antibiotics in the treatment of ventilator-associated pneumonia [4,14].

Three recent studies used the RIFLE (Risk - Injury - Failure - Loss - End stage renal disease) classification to determine CMS-associated nephrotoxicity [20,22,27]. The RIFLE criteria (Table 2) represent an extensively validated tool for evaluation of acute kidney injury, ranging from mild renal dysfunction to need for renal replacement therapy [30,31]. Hartzell et al. retrospectively reviewed 66 young adult patients who received intravenous CMS for at least 3 days [20]. Overall, 30 (45%) patients exhibited criteria for nephrotoxicity at the time of peak creatinine level (Risk: 13 patients; Injury: 10 patients; Failure: 7 patients). In 21% of the patients, CMS was stopped because of nephrotoxicity. No patient required renal replacement therapy. One month after the last CMS dose, criteria for Risk and Injury were still present in respectively 14 (28%) and 1 (2%) of 50 evaluable patients. In accordance with other studies [15,27,32,33], kidney injury was found to be related to the total cumulative dose and the duration of CMS therapy. Kwon et al. determined the incidence of CMS-associated kidney injury in 71 adult patients receiving CMS for more than 3 days [22]. Thirty-eight (53.5%) patients experienced nephrotoxicity (Risk: 11 patients; Injury: 10 patients; Failure: 17 patients). Compared with the study of Hartzell et al. [20], these patients were older, more severely ill, and also had chronic kidney disease or comorbidities predisposing them to renal toxicity. Cumulative dose of CMS was lower, probably because the dosage was more frequently modified for renal impairment. After discontinuation of CMS, renal function recovered completely in 16 (42%) patients. Cox regression analysis based on the cumulative dose of CMS identified four independent factors predicting acute CMS-induced kidney injury: male sex, concomitant use of a calcineurin inhibitor, hyperbilirubinemia, and hypoalbuminemia. The incidence of kidney injury increased with an increase in the number of risk factors. Hypoalbuminemia also was identified as an independent risk factor for CMS-induced nephrotoxicity in another study [21]. It is hypothesized that high serum levels of free colistin might enhance renal toxicity in patients with low albumin levels. Hypoalbuminemia also may reflect the severity of the underlying illness. Finally, DeRyke et al. retrospectively studied 30 patients treated with CMS for at least 48 h [27]. Nephrotoxicity was observed in ten (33%) patients (Injury: 3 patients; Failure: 5 patients; End-stage: 2 patients). Patients who developed nephrotoxicity were older, had more shock, and received excessive daily doses of colistin.

**Table 2:** RIFLE classification (serum creatinine and GFR criteria)

Category	Criteria
Risk (**R**)	Increased creatinine level × 1.5 or GFR decrease >25%

Injury (**I**)	Increased creatinine level × 2 or GFR decrease >50%

Failure (**F**)	Increased creatinine level × 3, GFR decrease >75% or creatinine level >4 mg/dL

Loss (**L**)	Persistent acute renal failure or complete loss of function for >4 weeks

ESKD (**E**)	ESKD for >3 months

GFR = glomerular filtration rate; ESKD = end-stage kidney disease

All studies using the RIFLE criteria reported a considerably higher incidence of CMS-induced nephrotoxicity [20,22,27]. A possible explanation may be the very high sensitivity of the RIFLE criteria, identifying acute kidney injury at creatinine values that are largely below the critical levels used to define renal failure (mostly above 1.3 to 2 mg/dL) in the other studies. However, it is striking that the studies reporting the highest incidence of colistin-associated nephrotoxicity [18,20,22,27] used products containing 150 mg of "colistin base activity." This has important implications for therapeutic dosing because 150 mg of colistin base corresponds with approximately 400 mg (or 5 million IU) of CMS. Given that many patients included in these studies had some degree of renal dysfunction at baseline or were treated for prolonged periods of time, it is possible that the observed nephrotoxicity was caused by "overdosing" with CMS. This is particularly obvious in the study of DeRyke et al. [27] where dose calculations based on actual body weight resulted in daily CMS doses of up to 25 million IU in some patients!

In summary, CMS has nephrotoxic effects but its potential to injure the kidney is probably overestimated particularly when very sensitive criteria (i.e., the RIFLE classification) are used. CMS-induced nephrotoxicity is mostly mild and reversible. Renal replacement therapy is occasionally required and permanent kidney damage is rarely seen. Still, deteriorating renal function remains an independent factor predicting treatment failure and increased mortality. Factors that may potentiate renal toxicity in an ICU setting, such as concomitant nephrotoxic medication, sepsis, shock, and hypoalbuminemia, should be adequately controlled. Rigorous application of recently highlighted measures designed to prevent kidney injury and to protect renal function in an ICU population remains warranted [34]. The observed association between total cumulative colistin dose and kidney damage suggests that shortening the duration of treatment for specific infections (e.g., pneumonia) could decrease the incidence of nephrotoxicity. It must be emphasized that dosage and frequency of colistin administration must be adjusted for serum creatinine levels and thus require close monitoring of renal function. Finally, the decision to stop colistin treatment on the basis of renal dysfunction must be weighed against the consequences of withholding a potentially life-saving antibiotic.

## Neurotoxicity

The interaction of colistin with neurons, which have high lipid content, has been associated with the occurrence of peripheral and orofacial paresthesias, visual disturbances, vertigo, mental confusion, ataxia, and seizures [32]. The most dreaded neurotoxic event, however, is neuromuscular blockade presenting as a myasthenia-like syndrome or as respiratory muscle paralysis producing apnea [35,36]. Potential triggers of neurotoxicity are hypoxia, concomitant medication (muscle relaxants, narcotics, sedatives, anesthetic drugs, and corticosteroids) and impaired renal function. The incidence of colistin-associated neurotoxicity reported in the literature before 1975 was approximately 7%, with paresthesias constituting the main event. Only sporadic cases of apnea were reported, typically in patients receiving colistin intramuscularly, suffering acute or chronic renal failure or treated with medications known to potentially induce respiratory muscle weakness [29]. More recent studies--all retrospective in design--did not observe a clear association between colistin treatment and neurotoxic events. Falagas et al. described four patients who had polymyoneuropathy during colistin treatment [6]. However, three patients already had neurological symptoms before colistin was started and in the one remaining patient, polyneuropathic symptoms subsided despite colistin was continued for 11 more days. Sabuda et al. reported four patients with varying neurological complaints [18]. All had developed significant renal dysfunction during treatment. Two patients had concomitant neurotoxic medication (gabapentin, baclofen, and tizanidine) or disorders (multifocal acute encephalopathy) that might have contributed to their neurological "distress" (respectively somnolence and vertigo). One patient with respiratory muscle weakness had received the equivalent of 13 million IU of colistin base per day for 19 days whilst experiencing a doubling of plasma creatinine levels. In a cohort of 115 patients, Cheng et al. identified four cases of potential colistin-induced neurotoxicity, including three patients with focal seizures and one patient with altered mentation [23]. These patients had normal kidney function but details about concomitant treatment or comorbidities were not given.

Diagnosis of neurotoxicity is mostly made on clinical grounds, making it difficult to discriminate between eventual colistin-induced neurotoxicity and the more frequently observed "critical illness polymyoneuropathy" in ICU patients. In only one study, electrophysiological measurement was performed in a limited number of patients who had received colistin for at least 7 days. Among these patients, 50% exhibited typical features consistent with critical illness polymyoneuropathy, but none had evidence of neuromuscular junction blockade [4]. Of note, no cases of clinically significant neurotoxicity were observed in a large group of patients with underlying neurological disease or disorders admitted to a neurosurgical ICU [11]. Finally, neuromuscular blockade was never seen in prospective studies evaluating CMS treatment [2-4,8,12,13,15].

## Optimization of colistin therapy in critical illness

The paucity of pharmacologic information regarding colistin administration in the critically ill highly impedes the creation of optimal dosing regimens that reconcile adequate antibacterial activity with minimal toxicity. Colistin pharmacokinetics are expected to be dramatically altered in critically ill patients, because they are frequently prone to large swings in distribution volume, fluctuations in renal clearance, and variable protein binding. Also, the antibacterial activity of colistin is attenuated in the face of high bacterial loads, as may be seen in pneumonia [37].

Data on colistin pharmacokinetics in critically ill patients with pneumonia and/or sepsis obtained by specific chromatographic assays became recently available [38-40]. The administration of CMS at a dose of 2 million IU [40] or 3 million IU [38,39] every 8 h resulted in maximum mean steady-state concentrations (C_max_) of colistin between 2.21 and 2.93 μg/mL. These findings are troublesome, because they indicate that currently prescribed CMS doses may be inadequate for treatment of infections caused by pathogens with minimal inhibitory concentration values in the upper range of the susceptibility breakpoint for colistin (2 μg/mL) and could induce the selection of resistant strains. Whether this has an impact on clinical cure and/or outcome is not clear. From the study by Plachouras et al. [39], it is obvious that it takes 2 to 3 days to reach the C_max _of colistin. These authors speculate that a loading dose of 9 to 12 million IU of CMS, followed by a maintenance dose of 4.5 million IU every 12 h would achieve the target C_max _faster with less frequent administration. It remains to be investigated whether this will lead to improved treatment efficacy without raising concern about toxicity. Moreover, a recent *in vitro *pharmacodynamic study in a *Pseudomonas aeruginosa *model showed that dosing regimens incorporating higher doses of colistin administered less frequently produced similar bacterial killing at the cost of a greater emergence of resistance than the conventional thrice-daily regimen [41].

## Conclusions

CMS is mostly prescribed for treatment of MDR *Acinetobacter baumannii *and *Pseudomonas aeruginosa. *Clinical cure rates are relatively high, especially when administered as monotherapy or in combination with a carbapenem. The dose varies considerably between studies but has become standardized over time to 9 million IU per day in patients with normal renal function.

CMS is potentially nephrotoxic but the incidence of kidney injury is probably overestimated by currently used criteria (e.g., the RIFLE classification) and may be influenced by manufacturer-dependent differences in dose recommendations. Although mostly mild and reversible, a decrease in kidney function must not be neglected, because it aggravates prognosis. The use of sensitive criteria to detect kidney injury could prove beneficial, because they may prompt clinicians to adequately address disease states, metabolic disorders, and medications that may enhance or precipitate colistin nephrotoxicity as well as encourage them to adapt CMS dosage or treatment duration in a timely manner.

Neurotoxicity does not seem to be a major adverse event accompanying colistin treatment. However, further studies must determine whether and how colistin interferes with underlying or ICU-acquired neurological disease (e.g., epilepsy, septic encephalopathy, critical illness polymyoneuropathy).

More research on colistin pharmacokinetics and pharmacodynamics in critically ill patients is urgently needed to guide adequate colistin dosing at the least toxicity.

## Competing interests

The authors declare that they have no competing interests.

## Authors' contributions

HS and RJ conceived and wrote the review. VVG and JT participated in literature search and selected appropriate articles. PMH participated in design, coordination, and writing. All authors read and approved the final manuscript.
